# Hypolipidemic and Antioxidative Effects of Aqueous Enzymatic Extract from Rice Bran in Rats Fed a High-Fat and -Cholesterol Diet

**DOI:** 10.3390/nu6093696

**Published:** 2014-09-16

**Authors:** Yu-Xin Wang, Yang Li, An-Min Sun, Feng-Jiao Wang, Guo-Ping Yu

**Affiliations:** 1 College of Science, Northeast Agricultural University, Harbin 150030, China; E-Mail: wyxin0612@126.com; 2 College of Food Science, Northeast Agricultural University, Harbin 150030, China; E-Mails: strive2011@126.com (A.-M.S.); fengjiaowang163@163.com (F.-J.W.); 3 Department of Respiratory Medicine, Daxing Hospital Affiliated to Capital Medical University, Beijing 102600, China; E-Mail: yamshang@163.com

**Keywords:** rice bran, aqueous enzymatic extract, hypolipidemic, antioxidant, HMG-CoA reductase

## Abstract

Purpose: The aqueous enzymatic extract from rice bran (AEERB) was rich in protein, γ-oryzanol and tocols. The aim of this study was to investigate the effects of AEERB on the regulation of lipid metabolism and the inhibition of oxidative damage. Methods: The antioxidant activity of AEERB* in vitro* was measured in terms of radical scavenging capacity, ferric reducing ability power (FRAP) and linoleic acid emulsion system-ferric thiocyanate method (FTC). Male Wistar rats were fed with a normal diet and a high-fat and high-cholesterol diet with or without AEERB. After treatment, biochemical assays of serum, liver and feces lipid levels, the antioxidant enzyme activity, malondialdehyde (MDA) and protein carbonyl were determined. Result: AEERB is completely soluble in water and rich in hydrophilic and lipophilic functional ingredients. AEERB scavenged DPPH• and ABTS^•+^ and exhibited antioxidant activity slightly lower than that of ascorbic acid in the linoleic acid system. The administration of AEERB reduced serum lipid levels and the atherogenic index compared with those of the hyperlipidemic diet group (HD). The administration of AEERB significantly lowered liver lipid levels, inhibited hepatic 3-hydroxyl-3-methylglutaryl CoA reductase activity, and efficiently promoted the fecal excretion of total lipids and total cholesterol (TC) (*p* < 0.05). Dietary AEERB enhanced antioxidant status in the serum, liver and brain by increasing the antioxidant enzyme activity of superoxide dismutase (SOD), catalase (CAT), and glutathione peroxidase (GSH-Px) and decreasing the content of MDA and protein carbonyl. Conclusions: The results indicated that AEERB might act as a potent hypolipidemic and antioxidant functional food.

## 1. Introduction

Hyperlipidemia is a risk factor for the development of cardiovascular diseases (CVD), including atherosclerosis (AS), coronary heart disease (CHD), and hypertension. CVD is the major cause of human death in China as well as other nations in the world [1]. Numerous studies have demonstrated that elevation in serum total cholesterol (TC) and low-density lipoprotein cholesterol (LDL-C) and reduction in high-density lipoprotein cholesterol (HDL-C) increase the risk of AS and CHD [2]. Oxidative damage initiated by free radicals is a major contributor to CVD development [3,4]. Scientists have become increasingly interested in functional foods because of the serious side effects of cholesterol-lowering drugs.

Rice is widely consumed globally, and rice bran (RB) is an important by-product of rice milling; RB contains the aleurone layer and some parts of the rice endosperm and germ. Recently, RB has attracted much attention because of its comprehensive nutritional and functional properties [5,6,7,8]. RB contains high valued protein, fat and bioactive phytochemicals with antioxidant and lipid-lowering properties including γ-oryzanol, tocols (tocopherols and tocotrienols) and flavonoid compounds. Although natural antioxidant components and nutritional proteins are abundant in RB, its potential application as a natural and raw material for the preparation of functional foods or nutraceuticals is limited because of the high insolubility of its protein, the integrity of the nutraceutical components of the product, its tendency to rancidity, and storage difficulty associated with it [9]. Previous studies have focused primarily on RB oil (γ-oryzanol and tocols) and functional protein or peptide, which were researched respectively, and several studies have demonstrated the hypolipidemic efficacy of RB oil in rodents [2,10,11,12] rabbits, primates and humans [13]. The aqueous enzymatic extract from rice bran has been reported rarely, and the studies only reported that rice bran enzymatic (endoprotease mixture) extract attenuated dyslipidemia and had an antioxidant capability [3,8,9]; the mechanism of the effect of rice bran enzymatic extract on cholesterol metabolism is not clear because measurement of fecal lipids and hepatic HMG-CoA reductase activity is lacking. There has not been a compositive evaluation of the antioxidant effect of RB enzymatic extract against the oxidative damage by antioxidant enzyme activities and the protein carbonyl content in hyperlipidemic rats.

Dietary change is the first treatment for improvement of metabolic syndrome symptoms [14,15]. We developed an aqueous enzymatic extract from rice bran (AEERB) that is completely soluble in water and rich in functional compositions—not only hydrophilic ingredients (including protein, polyphenols and flavonoid compounds), but also lipophilic ingredients (including γ-oryzanol, tocopherols and tocotrienols) [16], which could contribute to the treatment of chronic diseases caused by dyslipidemia and oxidative stress. The aim of this study was to evaluate the effects of AEERB on lipid metabolism (including serum, liver and fecal lipid levels, apolipoprotein A (ApoA), apolipoprotein B (ApoB) and lipoprotein (a) (Lp (a)) in serum and hepatic HMG-CoA reductase activity) in hyperlipidemic rats and the antioxidant capacity of AEERB* in vitro* and* in vivo*.

## 2. Materials and Methods

### 2.1. Materials

The RB was provided by the Great Northern Wilderness Agriculture Co., Ltd. (Heilongjiang Province, China). From Sigma-Aldrich Co., Ltd. (Shanghai, China), we obtained 2,2-diphenyl-1-picrylhydrazyl (DPPH), trypsin (1:250) and tocopherol and tocotrienol isomers, and 2,2ʹ-azino-bis (3-ethylbenzo-thiazoline-6-sulphonic acid) (ABTS) and γ-oryzanol were purchased from TCI (Tokoy, Japan); 2,4,6-tripyridyl-s-triazine (TPTZ) was obtained from Fluka (Buchs, Switzerland). Folin-Ciocalteau reagent was purchased from Solarbio Technology Co., Ltd. (Beijing, China). Standard of rutin was obtained from NICPBP (Beijing, China), and butylated hydroxytoluene (BHT) and ascorbic acid (AA) were purchased from Sinopharm Chemical Reagent Co., Ltd. (Shanghai, China). The other chemicals were of analytical grade.

### 2.2. Preparation and Composition of AEERB

AEERB was prepared according to an enzymatic process previously reported by our laboratory with slight modifications [16]. The RB was passed through a 40-mesh sieve and was modified by enzymatic hydrolysis by trypsin in a water bath with controlled temperature (56 °C) and pH of 7.9 (optimized by response surface methodology), using the pH-stat method. The processing of the product followed different procedures, including centrifugation, filtration, concentration and lyophilization. The final product is a brown powder that is completely soluble in water.

The chemical composition of AEERB was analyzed as follows: the total protein content was determined by the Kjeldahl method (a nitrogen conversion factor of 5.95); the total fat content was determined by the Soxhlet method; the reduced sugar content was determined by the Somogyi method [17]; the total phenolic content (TPC) was determined by the Folin-Ciocaltue method [18] and expressed as milligram gallic acid equivalents (GAE)/g AEERB (RB); the total flavonoid content (TFC) was determined by the method described by [19] and expressed as milligram rutin equivalents (RE)/g AEERB (RB); the γ-oryzanol content was determined by the method described by [20]; and the compositions and concentrations of tocopherol and tocotrienol isomers were determined by HPLC according to GB/T 26635-2011.

### 2.3. Antioxidant Potential of AEERB in Vitro

There are many methods to determine the antioxidant capacity, and each one has its limitations. Different antioxidant assay methods might yield different activity trends because of the reaction mechanisms [21,22,23]. The antioxidant activity of AEERB was evaluated by the following four different methods: (1) ferric reducing ability (FRAP) [24]; (2) DPPH• scavenging activity [25]; (3) ABTS^•+^ scavenging activity [26]; (4) linoleic acid emulsion system-ferric thiocyanate method (FTC) [27]. The lipid peroxidation inhibition (LPI) percentage (the content of the sample: 500 μg/mL) at 72 h was calculated as:

LPI (%) = [(1 − A_500 nm, sample_)/A_500 nm, control_] × 100 (1)

### 2.4. Animals and Treatment

The male Wistar rats (body weight 190–230 g, from the Laboratory Animal Center of Harbin Medical University in China) were conditioned to the environment of the animal house (temperature 21 ± 1 °C, relative humidity 55% ± 5%, under a 12 h light/dark cycle). The rats were allowed free access to commercial pellets (KeAoXieLi Feed Co., Ltd., Beijing, China) and tap water for 7 days before the experiment. After one week of acclimatization, the rats were randomly divided into three groups of similar average body weight (*n* = 10, each) and were given different dietary treatments. During the 42-day period, two control groups were fed a normal diet (ND) or a hyperlipidemic diet (HD) [28] (the diets compositions are shown in Table 1), and the treatment groups were fed the hyperlipidemic diet plus AEERB at 750 mg/kg body weight (BW) (AEERB) through gastric infusion. The rats in the ND and the HD groups were given oral administrations of saline. The body weights were measured every 3 days, and the daily food intake was recorded. The experiments were conducted according to the “Rules for Experimental Animals” guidelines published by Chinese government (Beijing, China). All experimental procedures were approved on 15 September 2013 by the ethical committee for laboratory animals of Northeast Agriculture University.

**Table 1:** Compositions of experimental diets.

Normal Diet	%	Hyperlipidemic Diet	%
Protein	20	Normal diet	78.8
Fat	4.5	Lard oil	10
Carbohydrates	52	Yolk powder	10
Fiber	4.3	Cholesterol	1.0
Moisture	9.1	Bile salt	0.2
Ash	8.0		
Calcium	1.3		
Phosphorus	0.8		

At the end of the experimental period, the rats were starved for 16 h and sacrificed by the removal of blood from the abdominal aorta. The serum samples were stored at −80 °C until analysis. After the blood collection, the livers were excised immediately and weighed. The entire liver was divided into several portions, and part of the liver tissue was immersed in 10% neutral formalin stock for pathology examination using H & E staining; the remaining liver tissue and brain tissue were frozen quickly in liquid nitrogen and stored at −80 °C until analysis. Feces were collected for the final 3 days of the experimental period.

### 2.5. Antioxidant Activity in Vivo

The antioxidant activities, including the total antioxidant capability (TAOC) as well as the SOD, CAT and GSH-Px activity and the MDA and protein carbonyl content in the serum, liver homogenates and brain homogenates were determined with enzymatic methods using corresponding commercial kits (Nanjing Jiancheng Bioengineering Institute, Nanjing, China).

### 2.6. Metabolic Parameters Analysis

The concentrations of TC, triglyceride (TG), LDL-C, HDL-C, ApoA, ApoB and Lp (a) in the serum lipids were measured using an Automatic Chemistry Analyzer (Hitachi 7600, Japan). The extraction of the lipids from the liver and fecal was based on the method of Folch and others [29], and the content was determined gravimetrically. The lipid concentrations were measured with corresponding commercially available kits [30] (Tissue total cholesterol assay kit, E1015; Tissue free cholesterol assay kit, E1016 and Tissue triglyceride assay kit E1013 from Applygen Technologies Co., Ltd., Beijing, China).

### 2.7. Hepatic HMG-CoA Reductase Activity Analysis

The hepatic HMG-CoA reductase activity was measured by ELISA methods with commercial kits (XinLe BioTechnology Co., Ltd., Shanghai, China).

### 2.8. Statistical Analysis

All the parameters were expressed as the mean ± SD. The results were statistically analyzed using one-way ANOVA followed by Tukey’s multiple comparison tests. The criterion for significance was *p* < 0.05. The analysis was performed with the Statistix 8.0 software package (Analytical Software, St. Paul, MN, USA).

## 3. Results and Discussion

### 3.1. Chemical Characterization of AEERB

The main components of AEERB and RB are presented in Table 2. Compared with RB, the enzymatic treatment markedly increased the content of the chemical compositions and bioactive compounds in AEERB except for that of the crude fat, which indicated that the enzymatic process had a significantly positive effect on the active compositions. As these data show, the main component of AEERB was protein (80.63% over RB) because the enzymatic hydrolysis reduced the size of the RB protein to peptide and free amino acid forms. Fat was the second major component (10.56%) and dissolved in this system due to the interaction with protein to form the water-soluble lipoproteins. The results were similar to that reported by Sungsopha and others [18]. In addition, the γ-oryzanol, β-tocopherol and α-tocotrienol content of the functional components in AEERB were much higher than RB, which were 2.8-fold, 2.1-fold and 2.6-fold, respectively, those of RB. This finding might be because of the effect of trypsin hydrolysis releasing free phenolic and fat-soluble components, which increased the levels of the bioactive compounds.

**Table 2:** Main compositions in AEERB and RB.

Composition	AEERB	RB
Protein (%)	26.10 ± 0.56 ^a^	13.43 ± 0.54 ^b^
Crude fat (%)	10.56 ± 0.3 ^b^	18.84 ± 0.09 ^a^
Reducing sugar (%)	8.43 ± 0.3 ^a^	1.47 ± 0.2 ^b^
TPC (mg GAE/g)	16.87 ± 0.006 ^a^	2.9 ± 0.1 ^b^
TFC (mg RE/g)	4.15 ± 0.07 ^a^	0.79 ± 0.06 ^b^
γ-Oryzanol (mg/g)	3.49 ± 0.1 ^a^	1.27 ± 0.08 ^b^
*Vitamin E (mg/kg)*		
α-Tocopherol	5.81 ± 0.28 ^a^	5.40 ± 0.71 ^a^
β-Tocopherol	0.85 ± 0.17 ^a^	0.40 ± 0.5 ^b^
γ-Tocopherol	1.19 ± 0.20 ^a^	1.30 ± 0.21 ^a^
α-Tocotrienol	4.42 ± 0.31 ^a^	1.7 ± 0.24 ^b^
γ-Tocotrienol	10.80 ± 0.42 ^b^	14.2 ± 0.40 ^a^
δ-Tocotrienol	1.30 ± 0.20 ^a^	1.20 ± 0.36 ^a^

The results are expressed as the means ± SD (*n* = 3). Different superscript letters in the same row indicate significant differences at *p* < 0.05. AEERB, the aqueous enzymatic extract from rice bran; RB, rice bran.

### 3.2. Antioxidant Potential in Vitro

The antioxidant potential of RB had been documented; it was concentrated in certain bioactive compounds of alcohol extracts or *n*-hexane extracts. In this study, the antioxidant activity of AEERB that was soluble in water was investigated by different principles. As summarized in Table 3, BHT and AA showed significantly higher antioxidant activity than that of AEERB on the FRAP assay and ABTS^•+^ scavenging activity; however, AEERB showed DPPH• scavenging activity similar to that of BHT and inhibition lipid peroxidation similar to that of AA. Among these methods, two types of radical scavenging activity analyses were used to evaluate the antioxidant capability of antioxidants with a hydrogen donor. Because of the relatively high level of polyphenol and flavonoid compounds in AEERB, AEERB had radical scavenging activity and the IC_50_ values of DPPH• scavenging activity and ABTS^•+^ scavenging activity were relatively close. These results indicated that AEERB had a noticeable antioxidant activity in fat-soluble systems and in water-soluble systems. AEERB might serve as a natural and safe antioxidative functional food or nutraceutical.

**Table 3:** Antioxidant capacity of AEERB* in vitro*.

Sample	Ferric Reducing Ability (mmol FeSO_4_·g^−1^)	DPPH• Scavenging Activity (IC_50_) (μg·mL^−1^)	ABTS^•+^ Scavenging Activity (IC_50_) (μg·mL^−1^)	LPI (%)
AEERB	3.15 ± 0.1 ^c^	98.87 ± 18.3 ^a^	103.83 ± 3.0 ^a^	22.72 ± 0.7 ^c^
AA	120.83 ± 0.6 ^a^	23.58 ± 5.5 ^b^	17.95 ± 0.6 ^b^	27.11 ± 0.3 ^b^
BHT	82 ± 0.9 ^b^	91.61 ± 6.3 ^a^	6.19 ± 0.5 ^c^	48.93 ± 0.8 ^a^

The results are expressed as the means ± SD (*n* = 3). Different superscript letters in the same column indicate significant differences at *p* < 0.05. AEERB, the aqueous enzymatic extract from rice bran; AA, ascorbic acid; BHT, butylated hydroxytoluene.

### 3.3. Effect on Body and Organ Weights

The growth parameters of the rats are presented in Table 4. The rats, fed with AEERB, had reduced adipose tissue weight and relative weight of liver compared with those in the HD group. The differences in the growth parameters between the AEERB group and the ND group were not significant, indicating that the administration of AEERB had no toxic effect at the experimental dose.

**Table 4:** Effect of AEERB on growth parameters in rats.

Growth Parameters	ND	HD	AEERB
Body weight gain (g/rat/day)	2.01 ± 0.88 ^b^	3.54 ± 1.15 ^a^	2.04 ± 1.11 ^b^
Food intake (g/day/rat)	24.02 ± 2.3 ^a^	18.19 ± 2.0 ^c^	19.75 ± 2.5 ^b^
Liver (g/100 g body weight)	3.22 ± 0.76 ^b^	3.89 ± 0.36 ^a^	3.54 ± 0.37 ^ab^
Epididymal adipose (g)	4.46 ± 1.33 ^b^	6.83 ± 2.53 ^a^	4.26 ± 0.95 ^b^
Perirenal adipose tissue (g)	0.57 ± 0.37 ^a^	1.00 ± 0.51 ^a^	0.76 ± 0.27 ^a^
Mesenteric (g)	2.06 ± 0.46 ^b^	3.05 ± 0.70 ^a^	2.36 ± 0.91 ^ab^

The results are expressed as the means ± SD (*n* = 10). Different superscript letters in the same row indicate significant differences at *p* < 0.05. ND, normal diet; HD, hyperlipidemic diet; AEERB, hyperlipidemic diet + 750 mg/kg/day of AEERB.

### 3.4. Antioxidant Activity in Vivo

As shown in Table 5, compared with the ND group, the antioxidant parameters of the serum TAOC, SOD, GSH-Px, CAT in the HD group were decreased by 32.94%, 70.02%, 16.42%, 26.41%, respectively, and the oxidative damage parameters of MDA and protein carbonyl content were concomitantly increased by 31.58% and 51.93%, respectively. Compared with the HD group, the treatment group showed significant antioxidant capabilities. The AEERB group demonstrated markedly higher TAOC and SOD activities than did the other groups and was 36.65% and 83.98% higher than in the HD group (*p* < 0.05), respectively. There was no significant difference in the serum GSH-Px, CAT and MDA content among the experimental groups. A similar trend of antioxidant status change was observed in the liver tissue and brain tissue (Table 6). The antioxidant parameters in AEERB group notably improved in the liver CAT and reduced in the liver protein carbonyl content compared to the HD group (*p* < 0.05). The antioxidant effect in the brain tissue was not significantly enhanced in AEERB group (*p* > 0.05), compared with that in the HD group. The antioxidant enzyme levels and activities (including SOD, CAT, GSH-Px) in the brain tissue are relatively low and enzymes metabolize slowly, which leads to a difficult recovery of the cumulative effect of oxidative damage. The brain tissue MDA and protein carbonyl were not significantly different in all the treatment groups. Previous studies have shown that hyperlipidemia could destroy the antioxidant defense system; decrease the activity of SOD, GSH-Px, and CAT; and elevate the lipid peroxide level [31]. Hypercholesterolemia could increase the cholesterol content of platelets, polymorphonuclear leukocytes and endothelial cells; lead to the formation of oxygen free radicals; and accelerate the process of lipid peroxidation. It is generally accepted that super-peroxide anions might be converted to H_2_O_2_ by SOD and then detoxified by CAT [32]. GSH-Px could transform toxic peroxide into a nontoxic hydroxy compound and promote the decomposition of H_2_O_2_ to protect the structure and the function of cell membranes from the damage and interference of peroxide. It is crucial to maintain enzyme activities to accommodate these oxidative stresses and reduce oxidative damage [33]. In this study, dietary AEERB remarkably enhanced the serum and liver antioxidant enzyme levels to decrease oxidative damage. MDA is the product of lipid peroxidation, which is an index of the oxygen free radical level. An increase in MDA leads to the promotion of AS caused by hyperlipidemia. Proteins are carriers of many important metabolites and nutrients and are the major focus of attack by free radicals. Free radicals change protein conformation by oxidative modification, which causes protein function loss and enzyme and receptor function decline, affecting normal physiological activities. The formation of protein carbonyl is an important indicator to determine oxidative damage to proteins. The evaluation of the antioxidant indicators of functional foods or nutraceuticals principally focuses on observing the activity changes of antioxidant enzymes and the extent of membrane lipid damage; the studies did not observe the proteins and nucleic acid damage. Our results demonstrated that the AEERB groups showed significantly higher antioxidant activity and reduced MDA and protein carbonyl content, which might be attributed to the antiradical activities of the bioactive and physiological active ingredients (including the polyphenol compounds and flavonoid compounds, γ-oryzanol and tocols) in AEERB known to act by free radical scavenging or chain-breaking mechanisms [34]. Our data is a significant complement to research on the hypolipidemic efficacy of RB.

**Table 5:** Effect of AEERB on serum antioxidant status.

Group	TAOC (U/mL)	SOD (U/mL)	GSH-Px (U)	CAT (U/mL)	MDA (nmol/mL)	Protein Carbonyl (nmol/mg protein)
ND	15.42 ± 1.62 ^a^	293.05 ± 20.09 ^a^	719.02 ± 87.75 ^a^	11.85 ± 2.49 ^a^	6.08 ± 1.87 ^a^	0.0518 ± 0.013 ^b^
HD	10.34 ± 1.75 ^b^	87.85 ± 22.36 ^c^	600.98 ± 173.00 ^a^	8.72 ± 3.26 ^a^	8.00 ± 1.44 ^a^	0.0787 ± 0.016 ^a^
AEERB	14.13 ± 0.85 ^a^	161.63 ± 26.57 ^b^	688.78 ± 124.28 ^a^	9.23 ± 3.44 ^a^	7.34 ± 1.87 ^a^	0.0596 ± 0.022 ^ab^

The results are expressed as the means ± SD (*n* = 10). Different superscript letters in the same column indicate significant differences at *p* < 0.05.

**Table 6:** Effect of AEERB on liver and brain antioxidant status.

Group	TAOC (U/mg protein)	SOD (U/mg protein)	GSH-Px (U)	CAT (U/mg protein)	MDA (nmol/mg protein)	Protein Carbonyl (nmol/mg protein)
*Live tissue*						
ND	1.83 ± 0.45 ^a^	109.17 ± 16.39 ^a^	117.34 ± 14.11 ^a^	45.21 ± 12.48 ^a^	1.03 ± 0.14 ^b^	1.78 ± 0.18 ^c^
HD	1.06 ± 0.25 ^b^	65.21 ± 10.60 ^b^	97.85 ± 17.23 ^b^	22.85 ± 6.77 ^b^	1.19 ± 0.12 ^a^	3.46 ± 0.50 ^a^
AEERB	1.40 ± 0.25 ^b^	97.63 ± 5.65 ^ab^	111.74 ± 11.01 ^ab^	41.81 ± 4.95 ^a^	1.08 ± 0.16 ^ab^	2.77 ± 0.77 ^b^
*Brain tissue*						
ND	1.09 ± 0.24 ^a^	55.20 ± 23.80 ^a^	66.33 ± 17.06 ^a^	11.83 ± 3.17 ^a^	3.52 ± 0.90 ^b^	2.28 ± 0.89 ^b^
HD	0.91 ± 0.13 ^a^	46.45 ± 18.09 ^a^	50.75 ± 27.42 ^a^	5.32 ± 1.85 ^b^	6.56 ± 1.35 ^a^	3.67 ± 0.53 ^a^
AEERB	1.04 ± 0.25 ^a^	52.24 ± 19.68 ^a^	63.13 ± 12.47 ^a^	6.79 ± 1.68 ^b^	5.52 ± 1.19 ^a^	3.29 ± 1.06 ^a^

The results are expressed as the means ± SD (*n* = 10). Different superscript letters in the same column indicate significant differences at *p* < 0.05.

### 3.5. Serum Lipid Analysis

This study explored the effect of AEERB on serum lipids in hyperlipidemia rats. Table 7 showed that there was a significant increase in the serum TC and TG in the HD group compared with the ND group, indicating that a high fat- and -cholesterol feed caused hyperlipidemia; and increased the risk of developing AS. Dietary AEERB countered the extent of hypercholesterolemia. The TC and TG in the AEERB group were lower than in the HD group. The serum lipid parameters of TC, TG, LDL-C and ApoB in the 750AEERB group decreased by 24.53%, 25.88%, 25.51%, 15.38% (*p* < 0.05, except ApoB), respectively, whereas HDL-C and ApoA increased by 32.76% (*p* < 0.05) and 22.73%, respectively, as compared with the HD group. The AEERB group showed more a notable effect in TC and TG than in the lipoproteins. No significant difference in ApoA and ApoB was observed between the HD group and the AEERB group (*p* > 0.05). Excessive LDL-C occurs in blood deposits in blood vessel walls and becomes a major component of AS plaque lesions. HDL-C facilitates the translocation of cholesterol from the peripheral tissues such as arterial wall tissue to the liver for catabolism [32,35]. Decreased TC, TG and LDL-C and increased HDL-C could lower the risk of developing CHD. The AI_1_ and AI_2_ atherogenic indexes express the risk extent of AS. A lower index value of index indicates a lower extent of AS. This study showed AI_1_ and AI_2_ were significantly reduced in the AEERB group compared to the index levels in the HD group (*p* < 0.05). Our data clearly indicated that administration of AEERB lowered cholesterol and reduced the risk of AS and CHD; AEERB might be used in the treatment of AS. Lp (a), an independent risk factor for CHD, is predominantly synthesized in the liver; its major function is to prevent the dissolution of blood clots and to promote the formation of AS. Angina pectoris, myocardial infarction, and cerebral hemorrhage are closely related to increased Lp (a) levels. In this experiment, the Lp (a) of the AEERB group was much lower than that of the HD group (an 18.09% reduction, *p* < 0.05). The positive effect of AEERB on apolipoprotein suggested that apolipoprotein was regulated by AEERB to combine with a corresponding receptor as the ligand, take part in lipoprotein metabolism and return lipid metabolism to normal, thereby playing a role in regulating lipid levels and lipoprotein metabolism disorders.

**Table 7:** Effect of AEERB on serum lipids in rats.

Serum Lipid Parameters	ND	HD	AEERB
TC (mmol/L)	1.52 ± 0.08 ^c^	2.65 ± 0.17 ^a^	2.00 ± 0.12 ^b^
TG (mmol/L)	0.50 ± 0.22 ^b^	0.85 ± 0.16 ^a^	0.63 ± 0.04 ^b^
HDL-C (mmol/L)	0.87 ± 0.06 ^a^	0.58 ± 0.06 ^c^	0.77 ± 0.09 ^b^
LDL-C (mmol/L)	0.51 ± 0.12 ^c^	0.98 ± 0.11 ^a^	0.73 ± 0.10 ^b^
ApoA (g/L)	0.039 ± 0.01 ^a^	0.022 ± 0.01 ^b^	0.027 ± 0.01 ^b^
ApoB (g/L)	0.026 ± 0.01 ^b^	0.039 ± 0.01 ^a^	0.033 ± 0.01 ^ab^
Lp (a) (mg/L)	1.37 ± 0.23 ^b^	1.88 ± 0.23 ^a^	1.54 ± 0.14 ^b^
AI_1_	0.75 ± 0.15 ^c^	3.59 ± 0.48 ^a^	1.65 ± 0.43 ^b^
AI_2_	0.58 ± 0.16 ^c^	1.71 ± 0.27 ^a^	0.97 ± 0.23 ^b^

The results are expressed as the means ± SD (*n* = 10). Different superscript letters in the same row indicate significant differences at *p* < 0.05. AI_1_, atherogenic index was calculated as (TC − HDL-C)/HDL-C; AI_2_, antiarterial hardness indices were calculated as LDL-C/HDL-C.

In addition, combined with the results of the antioxidant experiments, it was clear that serum TC, TG, and the atherogenic index were negatively correlated with the antioxidant indicators (TAOC, SOD, CAT, GSH-Px) and positively correlated with the MDA and protein carbonyl content. The lipid levels in the hyperlipidemic rats and their oxidation-antioxidant levels were shown to be closely related and mutually promotive. AEERB showed consistency in its lipid-lowering and antioxidative effects and had significant physiological lipid-lowering and antioxidative activities.

### 3.6. Liver Lipid Analysis

The liver lipid profile exhibited a similar tendency as that of the serum lipid profile. As shown in Table 8, the cholesterol-and fat-enriched diet promoted the accumulation of liver lipids, and the total liver lipids, TC, free TC, cholesterol ester and TG content in the HD group were higher by 1.66-, 1.40-, 1.32-, 1.46-, and 1.22-fold, respectively, than those of the ND group (*p* < 0.05), whereas the consumption AEERB showed an effect on reducing the lipid levels. Except for the free cholesterol, the lipid parameters of the AEERB group were obviously lower than those of the HD group (*p* < 0.05). This result was similar to that of a previous study regarding the effect of RB oil on the regulation of liver TC and TG [30,36]. Temel and others [37] reported that reduced hepatic cholesterol ester content caused an inhibition of cholesterol absorption. Decreased hepatic free cholesterol available after acyl coenzyme A-cholesterol acyltransferase (ACAT) inhibition is very rapidly directed for elimination in bile directly or after conversion to bile acids [38]. The lower hepatic free cholesterol and cholesterol ester storage in the AEERB groups might be the main factor that could effectively inhibit cholesterol absorption.

**Table 8:** Effect of AEERB on liver lipids in rats.

Group	Total Lipids (%)	TC (μmol/g)	Free Cholesterol (μmol/g)	Cholesterol Ester (μmol/g)	TG (μmol/g)
ND	33.46 ± 11.51 ^b^	67.33 ± 7.46 ^b^	28.54 ± 1.63 ^b^	38.79 ± 7.90 ^b^	58.23 ± 6.04 ^b^
HD	55.46 ± 26.20 ^a^	94.21 ± 2.83 ^a^	37.60 ± 6.74 ^a^	56.61 ± 6.73 ^a^	71.15 ± 5.5 ^a^
AEERB	15.47 ± 6.61 ^b^	70.01 ± 8.49 ^b^	33.68 ± 2.69 ^a^	36.32 ± 9.76 ^b^	41.40 ± 8.38 ^c^

The results are expressed as the means ± SD (*n* = 10). Different superscript letters in the same column indicate significant differences at *p* < 0.05.

### 3.7. Fecal Lipid Analysis

**Figure 1:**
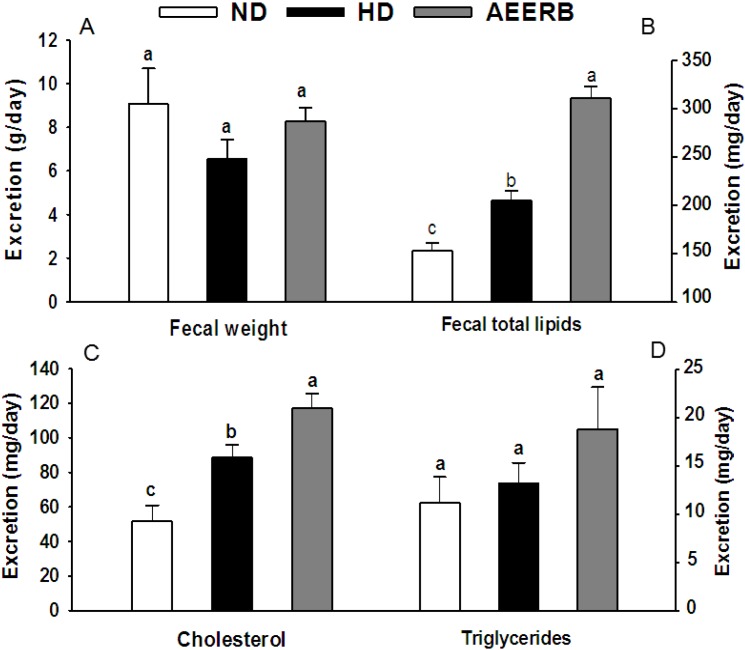
Fecal weight (**A**); total lipids (**B**); cholesterol (**C**) and triglycerides (**D**) of the experimental rats. The values are the mean ± SD (*n* = 3). Different superscript letters indicate significant differences at *p* < 0.05. ND, normal diet; HD, hyperlipidemic diet; AEERB, hyperlipidemic diet + 750 mg/kg/day of AEERB.

The fecal excretion and lipid levels are shown in Figure 1. The fecal excretion of the HD group was significantly reduced compared with the ND group. The fecal total lipids, TC and TG content in the AEERB group were higher than the levels in the ND group and the HD group, in which the total lipid and TC content showed the largest increases (*p* < 0.05). There was no significant difference in the fecal excretion and TG content in the experimental groups (*p* > 0.05). The fecal TC was considered another marker for the metabolism of cholesterol. In the intestinal mucosa, phytosterols, particularly sitosterol, could compete with and inhibit cholesterol absorption. There was high level of γ-oryzanol in AEERB. Thus, we hypothesize that the increase in the fecal lipid levels in the AEERB group might be because of the inhibition of cholesterol absorption in the intestinal tract.

### 3.8. Hepatic HMG-CoA Reductase Activity

**Figure 2:**
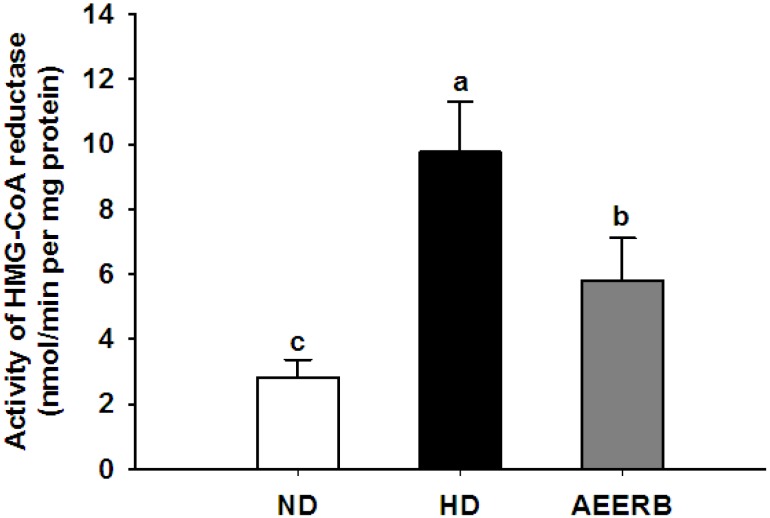
Activity of hepatic HMG-CoA reductase. The values are the means ± SD (*n* = 6). Different superscript letters indicate significant differences at *p* < 0.05. ND, normal diet; HD, hyperlipidemic diet; AEERB, hyperlipidemic diet + 750 mg/kg/day of AEERB.

The liver is the main organ of endogenous cholesterol biosynthesis, and HMG-CoA reductase is the key rate-limiting enzyme in the biosynthesis pathway of isoprenoids and cholesterol. We measured the activity of HMG-CoA reductase to determine whether the decrease in the liver cholesterol level was because of the inhibitory effect of this enzyme (Figure 2). The data showed that HMG-CoA reductase activity in the HD group was higher than in the ND group (2.46-fold); this result was in agreement with the findings by Mohammad and others [39]. As expected, the HMG-CoA reductase activity in the AEERB group significantly decreased, and was reduced by 40.59% compared with the HD group. Our results showed that the consumption of AEERB decreased the cholesterol levels by suppressing hepatic HMG-CoA reductase activity, likely through a posttranscriptional mechanism that is similar to the mechanism of the oxysterols. The ability of AEERB to reduce hepatic HMG-CoA reductase activity might be associated with abundant tocols, which corresponds with previous reports [2,39,40,41].

### 3.9. Pathological Examination

**Figure 3:**
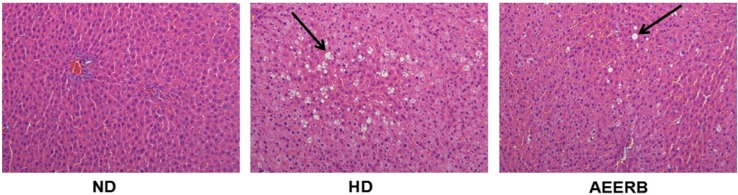
Pathological examination of the rat livers. The liver section was stained using H & E at 200×. ND, normal diet; HD, hyperlipidemic diet; AEERB, hyperlipidemic diet + 750 mg/kg/day of AEERB. Arrow indicated the lipid droplets.

The pathological examinations of liver sections of various groups are shown in Figure 3. The results indicated that AEERB could effectively inhibit liver fatty infiltration or steatosis and reduce the formation of the lipid droplets. AEERB could reduce hepatic lipid deposition to prevent the occurrence of fatty liver.

## 4. Conclusions

Cholesterol homeostasis is precisely controlled by the regulation of endogenous cholesterol synthesis and the absorption of exogenous cholesterol. We concluded that the hypolipidemic mechanisms of AEERB could be elucidated as follows: first, AEERB inhibits the absorption of cholesterol (phytosterols and γ-oryzanol) and enhances their excretion in feces; second, AEERB effectively reduces endogenous biosynthesis (tocols) by inhibiting the hepatic HMG-CoA reductase activity. The precise hypolipidemic mechanisms remain for detailed investigation in the future because this study did not include research on the excretion of bile acids and the relative expression of cholesterol and lipoprotein metabolism-related genes in the hepatic mRNA. This study suggests that AEERB, given its antioxidant and hypolipidemic activity, could be an excellent source for functional foods or nutraceuticals.
